# LCoMotion – Learning, Cognition and Motion; a multicomponent cluster randomized school-based intervention aimed at increasing learning and cognition - rationale, design and methods

**DOI:** 10.1186/1471-2458-14-967

**Published:** 2014-09-18

**Authors:** Anna Bugge, Jakob Tarp, Lars Østergaard, Sidsel Louise Domazet, Lars Bo Andersen, Karsten Froberg

**Affiliations:** Centre of Research in Childhood Health, Department of Sports Science and Clinical Biomechanics, University of Southern Denmark, Campusvej 55, 5230 Odense M, Denmark; Department of Sports Medicine, Norwegian School of Sport Sciences, Oslo, 0806 Norway

**Keywords:** Cognition, Academic achievement, Physical activity, Children, Adolescents, School-based intervention, Fitness, RCT

## Abstract

**Background:**

The aim of the study; *LCoMotion – Learning, Cognition and Motion* was to develop, document, and evaluate a multi-component physical activity (PA) intervention in public schools in Denmark. The primary outcome was cognitive function. Secondary outcomes were academic skills, body composition, aerobic fitness and PA. The primary aim of the present paper was to describe the rationale, design and methods of the LCoMotion study.

**Methods/Design:**

LCoMotion was designed as a cluster-randomized controlled study. Fourteen schools from all five regions in Denmark participated. All students from 6^th^ and 7^th^ grades were invited to participate (n = 869) and consent was obtained for 87% (n = 759). Baseline measurements were obtained in November/December 2013 and follow-up measurements in May/June 2014. The intervention lasted five months and consisted of a “package” of three main components: PA during academic lessons, PA during recess and PA homework. Furthermore a cycling campaign was conducted during the intervention period. Intervention schools should endeavor to ensure that students were physically active for at least 60 min every school day. Cognitive function was measured by a modified Eriksen flanker task and academic skills by a custom made mathematics test. PA was objectively measured by accelerometers (ActiGraph, GT3X and GT3X+) and aerobic fitness assessed by an intermittent shuttle-run test (the Andersen intermittent running test). Furthermore, compliance with the intervention was assessed by short message service (SMS)-tracking and questionnaires were delivered to students, parents and teachers.

**Discussion:**

LCoMotion has ability to provide new insights on the effectiveness of a multicomponent intervention on cognitive function and academic skills in 6^th^ and 7^th^ grade students.

**Trial registration:**

Clinicaltrials.gov: NCT02012881 (10/10/2013)

## Background

Prevalence of obesity and overweight is increasing among children and adolescents throughout the world [1]. At the same time it has been shown that physical activity (PA) and aerobic fitness have decreased particularly in the less fit pediatric population [2–4]. Taken together, the health consequences of these trends may be a future adult population with high prevalence of obesity and metabolic abnormalities [5].

PA has been suggested as an important factor in prevention of both obesity and lifestyle diseases [5–7]. Schools provide an advantageous setting for encouraging a healthy lifestyle among children and for enhancing PA [8], since school interventions can reach children from all risk groups [9], while stigmatization of children at risk likely is avoided. Previous school-based intervention studies addressing different health related outcomes have been inconclusive regarding results on body composition [10], PA [11–13] and aerobic fitness [11, 13]. Even though many of the school-based intervention studies found some improvements in health parameters, there still seems to be a lack of willingness of parents, teachers and politicians in many countries to prioritize PA during the school day. However, recent research has shown that PA and a high aerobic fitness level are associated not only with metabolic health, but also with improved cognitive function and academic skills [14–18]. If these results could be confirmed and expanded, it might change the priorities in different school systems towards more PA during the school day. Childhood is probably the most important time of life for development of cognitive skills, and given the assumed positive association with PA, it emphasizes the importance of early intervention to decrease inactivity and/or improve PA. On this basis, the Danish government has decided that every child in public schools should have 45 min of PA every school day starting from summer 2014 [19]. Some school-based interventions have already been conducted with the aim of investigating the effect of increased PA on cognitive function and/or academic skills [20–24]. Some of these studies were, however, small-sized [22–24], the analyses of the effect on academic skills were only performed on a sub-sample of the participants [21], or the study was not conducted as a randomized controlled trial [24]. With these limitations kept in mind, there seems to be a positive effect or at least no negative effect of taking time from academic subjects and directing it towards more PA. However, the most effective way to compose school-based interventions is still unknown. One challenge is to design an intervention which is effective in terms of improving health and cognitive function of children, while at the same time possible to implement in large scale school settings.

The overall objective of the present study; *LCoMotion – Learning, Cognition and Motion,* was therefore to evaluate the effect of a school-based PA intervention aiming at increasing PA to a total of 60 min per school day. LCoMotion is the first large school-based PA-intervention in Denmark assessing cognitive function and academic skills, as well as body composition, aerobic fitness and PA. The primary aim of the present paper is to describe the rationale, design and methods of the LCoMotion study.

## Methods/Design

### Study design

LCoMotion is a cluster randomized controlled intervention study including students from the 6^th^ and 7^th^ grade at public schools in Denmark (12-14 years old). The clusters consisted of participating schools. Schools were randomly allocated either to the intervention or control group. After the follow-up measurements all control schools received the same intervention "package" as the intervention schools.

### Recruitment and randomization

**Figure 1:**
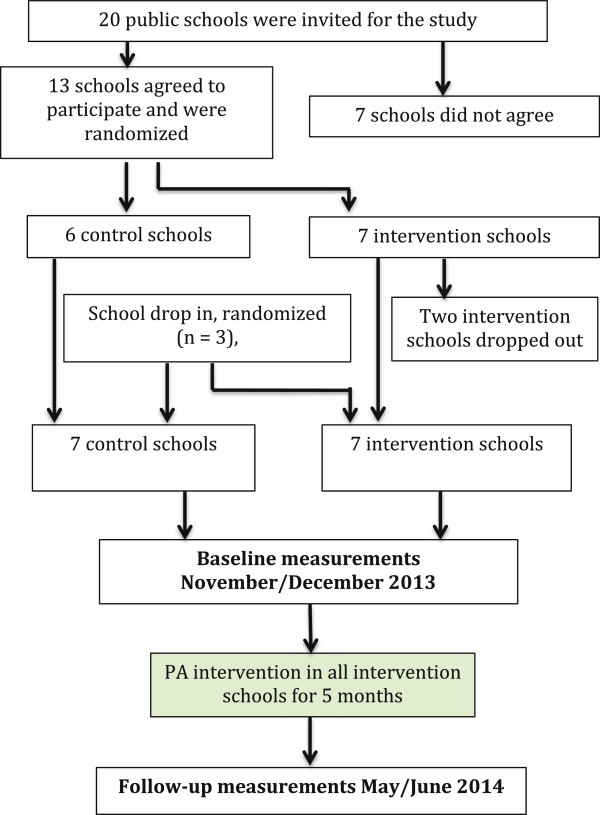
**Flowchart of schools in LCoMotion.**

A complete chart illustrating the recruitment and randomization process is shown in Figure 1. Baseline measurements were done in November/December 2013, follow-up measurements in May/June 2014. Data analysis has not yet been completed. All schools participating in a primary-school project about PA and health (Styr på Sundheden [25]) were invited to participate in LCoMotion. Out of 20 schools, 13 schools accepted the invitation. Schools were randomly assigned to receive either the intervention "package" or serve as controls. Two intervention schools withdrew the acceptance before the start of the study, but after the randomization. Additionally three schools not originally enrolled in the study were invited from local networks and randomized. In total 14 schools from all five main regions of Denmark were recruited for the study. From these schools all students in 6^th^ and 7^th^ grade classes (n = 869) were invited to participate in LCoMotion by a letter describing the study, the measurements, the randomization procedures and the consent form. All students, parents and teachers were then invited to a meeting at the school, where the scientific background and a full description of the study was given. Eighty-seven percent of the parents gave consent for their child to participate in the study (n = 757). The study was approved by the ethics committee of the region of Southern Denmark (S-20130104) and registered at clinicaltrials.gov (NCT02012881) (10/10/2013).

### Intervention components

The intervention "package" in LCoMotion consisted of three main components:

1. *Active Learning*. Participating classes’ primary teachers, and other interested teachers received supplementary training in delivering activities involving PA during academic subjects. The supplementary training consisted of a 3 hours course on one day. Focus was on practical tools to incorporate PA in the different subjects. All teachers were expected to register these activities using an "activity watch" hanging in each class room. The primary teacher of each class reported the total number of minutes used on "active learning", as well as the number of times that the teacher initiated PA in academic subjects, to the research team every week by text messages (see Outcome measures).
2. *Physically active recesses.* Teachers and a selected group of students received supplementary training on how to conduct moderate to vigorous physical activities in recess with their students or peers. This course was partly with teachers and students together and partly with the two groups separated. The course was delivered on one day on each school and lasted 4 hours. Furthermore, all intervention schools received a bag with equipment for these activities, e.g. different types of balls, Frisbees, pins etc.
3. *PA home work.* Students received a booklet with different activities lasting about 10 minutes each. They had to select one activity each day and register how many days they carried out an activity. Each month the teachers collected these booklets and distributed new ones with other activities. In the end of the intervention, there was a competition between classes of having most activities done.

Furthermore, during the third month of the intervention, all intervention classes received materials regarding a cycling commuting competition in the form of posters in the classrooms and information via the school’s intranet. All intervention classes competed for the highest frequency of cycle commuting during two weeks. Results were registered by the short message service (SMS) tracking system (see below).

All participating schools (principals and involved teachers) made an oral agreement with the research staff about endeavor to ensure that all participating student had 60 minutes of PA on all school days during the intervention. No guidelines on how to distribute the 60 minutes of daily PA were given. The practical implementation was therefore completely decided and administrated by the teachers and schools. This is also the case in the new Danish school reform, which intends to implement 45 minutes of PA every day in the school setting to all students [19].

### Data collection procedures and staff training

Prior to both baseline and follow-up testing all research personnel was trained by members of the research team on the assessment of mathematics skills, cognitive function, cardiovascular fitness, anthropometrics and self-assessment of pubertal status. Pilot tests of the flanker task and the mathematics test were conducted (see Outcome measures).

Data collections were performed at the participating schools in a gym and in the classrooms, except for the flanker task, which was performed in smaller rooms with a maximum of four students present at the same time.

### Outcome measures

#### Primary outcome

##### Cognitive function

Cognitive function was evaluated using a computer-based modified Eriksen flanker task [26]. An arrow pointing to the right “>” will require a right-handed response and an arrow pointing to the left “<” will require a left-handed response. These two response-eliciting stimuli are flanked by an array of other arrows that are congruent (>>>> > or < <<<<), or incongruent (>><>> or <<><<) to the centrally placed target stimulus. The incongruent condition is found to cause a delay in response time as well as a decrease in accuracy relative to the congruent condition, mainly because it requires greater amounts of inhibitory control to overwrite the stimuli induced by the distracting flankers. The congruent and incongruent conditions were randomly introduced. Participants completed one practice block of 20 trials to train the participants in what was expected, and to identify any problems they had in understanding and performing the test. If this test was not satisfactory, another practice block was completed, and this was repeated until the tester was certain whether the participant had understood the task correctly. Subsequently two blocks consisting of 75 trials each were presented for 120 milliseconds (msec) followed by a response window of 1350 msec. A random inter-stimulus interval of 1250 – 1550 msec separated each trial and a break of 30 seconds separated each block of trials. The entire test could be completed within 8 minutes. Students were instructed to respond as quickly and as accurately as possibly. The outcomes for the test were reaction time (RT) to the stimulus of the arrow on screen (msec) and accuracy (percentage of possible trials where the correct response was selected). Calculation of interference scores (incongruent – congruent) for both RT and accuracy will allow for interpretation of the flanker effect as the difference in speed and accuracy of information processing during the congruent and the incongruent trials [27]. Performance on the flanker task is unknown in a general population of adolescents in the midst of puberty. The progression through puberty could be of importance (though a similar progression in the control group and the intervention group would be expected) to both outcome measures (accuracy and RT) [27]. A pilot study was therefore conducted prior to the baseline testing on the study age group, and the flanker task was accordingly adjusted, in order to elicit a response accuracy of around 70 – 80% in the incongruent trials.

### Secondary outcomes

#### Academic skills

Academic skills were assessed by a mathematics test consisting of 50 questions of varying difficulty matching the requirements of 6^th^ and 7^th^ grade curriculum, respectively. No aids were permitted in the mathematical skill testing. A modified version (only numbers were changed) was administered at follow-up measurements. Students were allowed 45 minutes to complete the tests. The math tests were scored by the research staff. A pilot study was conducted and the mathematics skill tests were validated against a standardized math-test often used in the Danish school system to asses mathematics skills at the individual level in different age-groups [28]. A correlation coefficient of 0.87 (p < 0.001) was found between the standardized and the applied test.

### Physical activity (PA) measurements

PA levels of participants were assessed by accelerometry before and during the intervention. GT3X and GT3X + devices (ActiGraph LLC, Pensacola, FL, USA) were distributed by research staff to students at schools with the instruction to wear the devices on the right hip every day during a seven day period. Devices were distributed on Mondays or Tuesdays during November/December 2013 (before intervention) and during March 2014 (third month of the intervention). Accelerometer type was randomly selected at the student level and assessment periods were balanced between control and intervention schools. Due to a shortage of devices one intervention school did not receive accelerometers, as this school entered the study just prior to baseline testing. Furthermore, accelerometers were only distributed in four of five, four of six and three of four classes at another three schools.

Outcomes for PA levels will be mean counts/min during all assessed minutes as well as time (minutes) spent in different intensity domains (sedentary, light, moderate, vigorous PA). These parameters can furthermore be investigated in segments of the day (school time, recess, and leisure-time) as class-specific time tables were collected at both assessment periods. These segments were all components in the LCoMotion intervention and it will thus be possible to investigate whether the intervention caused any changes in PA during the different segments of the day.

### Cardiorespiratory fitness

Aerobic fitness was measured with the Andersen intermittent running test [29]. Two parallel lines 20 m apart were made on the floor. Subjects ran from one line to the other. They had to touch the line on the floor with one hand, turn around and run back. Music signaled 15 seconds of running and 15 seconds of rest. Participants had to stop immediately at the signal. This procedure was followed for 10 minutes giving an overall running time of 5 minutes. Subjects were told to run as fast as they could in order to cover the longest possible distance. This distance in meters was the test result, which has been validated against direct measures of maximal VO_2_-uptake [29, 30]. The test has also been found to have a fairly high reliability [30].

### Anthropometric measures and puberty assessment

Body height was measured without shoes to the nearest 0.5 cm using Harpenden stadiometer (West Sussex, UK). Body mass was measured in light clothing to the nearest 0.1 kg using an electronic scale (Tanita BWB-800, Tokyo, Japan). Waist circumference was measured to the nearest 0.5 cm with an anthropometric tape, at the level of the umbilicus. Sexual maturation was assessed by self-report using a scale of pictures of pubic hair for both sexes and of breast and genital development for girls and boys, respectively, according to the development divisions presented by Tanner [31]. Furthermore, girls had to report if they had started having menstrual periods.

### Short message service (SMS)-tracking quantifying the intervention elements

Every second week for the entire intervention period information on compliance with the intervention elements was collected using SMS-tracking on mobile phones (Automatic text messaging). Students at intervention schools received a message every second Friday asking how many times the last week;

- they had participated in programmed recess activities
- their teachers had initiated physical activities in the academic subjects
- they had conducted their PA homework.

Once a month both intervention and control students were asked about bicycling to and from school the past week.

Main class teachers in all intervention classes were sent two questions each week;

- how many minutes the class had been physically active (both as planned recess activities and in academic subjects) in the past week following the “activity watch”
- how many times they themselves had initiated physical activities in academic subjects

For all messages both for students and teachers, if they were not answered, a follow-up message was send after 24 hours and again after 72 hours. If the message was not answered after that, or answered in text or other invalid ways, students or teachers were contacted by the research team by phone to clarify facts.

### Questionnaires

Three different questionnaires were administered; one for participating students, one for the parents or guardians, and one for main class teachers in all participating classes. All questionnaires were paper based.

The questionnaire for students contained a total of 29 questions and was administered on the test day at the school or by the teachers one or two days after the test day. The questions covered activities in recess, self-assessed school performance, relations to classmates, teachers, friends and family, motivation for school, PA in leisure time, sedentary behavior, mode of transportation, sleeping habits, diet (short), smoking habits and alcohol consumption. These items were categorized into four domains (school time, intrinsic factors, leisure-time activities and dietary habits). The questionnaire for parents contained 29 questions on their child’s health including diagnosed syndromes or illnesses, specifically mental illnesses and need for special education. Questions on ethnicity, household income and education, and parental assessment of the child’s academic skills were also included in the questionnaire. The parent questionnaire was delivered to the students on the test day and brought home to the parents with a pre-paid return envelope. Only one parent or guardian for each student was requested to answer the questionnaire. After 3-4 weeks all parents were reminded to fill out and return the questionnaire by a message on the school’s intra-net. Those parents who had not returned the questionnaire after 6 weeks had a new questionnaire resent. The majority of questions in both students’ and parent’s questionnaires had previously been used in other (inter)national surveys about children and adolescents’ health and PA habits (e.g. [32–34]).

Questionnaire for main class teachers was delivered by the end of the intervention, but before the follow-up testing and contained questions regarding the amount of planned activities during recesses and the amount of physical activities during academic subjects. This was done in order to compare the quantity of these elements, as the SMS-tracking is only used for quantifying intervention elements in the intervention schools (except for bicycling which was assessed in both intervention and control schools).

#### Statistical considerations

Sample size calculation was done prior to recruitment of schools. Conventional two-sided levels of statistical power (0.8) and level of significance (0.05) were used. Calculation was done based on expected differences between intervention and control group in one of the primary outcomes; accuracy in the flanker task. An intervention with the duration of the LCoMotion intervention was hypothesized to result in an effect size of approximately three percent points (personal communication with Professor CD Hillman about effect size and standard deviations). To lower the assumed cluster effect schools were grouped based on their grade point average from the final examination 2011/2012. Assuming a cluster effect of 0.01 and an average number of students per school of 80, the calculations showed that a minimum of 11 schools (5 control and 6 intervention schools) were required, with a total number of 680 students. Analyses were performed with statistical software STATA version 13.

The effect of the intervention will be tested using multilevel mixed effects models adjusted for relevant possible confounding factors, e.g. sex, age, baseline value of the variable, family socioeconomic status etc. Because of the cluster structure of data, schools and classes will be included as random effects in the analyses.

### Baseline participation

Table 1 presents the response/participation of all participants with consent at baseline. The lowest rate is found for accelerometer data, since not all participants were involved in this measurement and not all who were, had a valid measurement (of at least one day with 10 hours). 47 participants were absent in the day of testing at baseline.

**Table 1:** **Response rates (%) for all consenting participants**

Variables	Intervention schools	Control schools	All schools combined
	(n = 234)	(n = 525)	(n = 759)
Anthropometrics (height, weight, waist circumference)	89%	91%	91%
Pubertal status	89%	90%	89%
Cognitive test	93%	93%	93%
Mathematic test	94%	90%	92%
Fitness test	88%	81%	83%
Student questionnaire	95%	89%	91%
Parental questionnaire	89%	88%	88%
Accelerometer*	89 (73)%	84 (72)%	86 (72)%

*Not all students did accelerometer measurements for assessment of physical activity. In total accelerometers were distributed to 84% of all students (n = 193 and n = 445 in intervention and control schools, respectively). Participation rate is calculated from returned accelerometers with at least one valid day of measurements (≥10 hours) relative to total number of accelerometers distributed. Numbers in brackets show the percentages of all participants with consent.

## Discussion

There is a lack of well-designed properly powered school-based intervention studies regarding the effect of PA on cognitive function and academic skills. The present study has the possibility to add significantly to the scientific knowledge in the field. The study has several strong points. First, the intervention was designed to be easily adapted to larger scale interventions. The entire intervention was conducted in a “real-life setting” by the school staff; teachers, principals and pedagogues, with support from scientific experts. Courses for teachers and students were delivered by a permanent actor in the field “Danish School Sports”, who has a long history of giving courses and information to Danish schools both in regard to recess activities, school sport and PA in academic subjects [35]. Finally, the cost of the intervention was relatively small, which makes it suitable to implement in larger populations.

Also, the quality of applied methods is a strength. The test for the primary outcome, cognitive function measured by the flanker task, is modified according to the specific age group in the study and chosen in dialogue with experts in the field (personal communication with CH Hillman).

To our knowledge, only a few studies have so far reported use of the flanker task in a large school setting [36, 37]. The majority of previous publications reporting use of the flanker task have used smaller sample sizes [23, 38], or been conducted on older individuals (e.g. [39]). It will thus be of interest to assess whether the present intervention can result in any differences in test results, and also to assess whether performance on the flanker task can be predicted from PA, fitness or body composition in a large general population of school-children.

Measuring PA is a notoriously difficult task. The combination of applied methods; accelerometry, questionnaires and sms-tracking taken together is considered a strength. A combination of methods is considered as the optimal solution for measuring PA in large cohort studies [40]. In that way, some of the problems in accelerometry measurements can be overcome; e.g. lack of intensity precision during bicycling and no measurements during water activities can be solved by asking question about these specific activities in questionnaires and by SMS. Accelerometry allows analysis of specific time periods such as recess or academic lessons (when coupled to school timetables). Also the use of SMS-tracking is a strength, as this method introduces a shorter recall time and thereby improves the validity of data and therefore is less exposed to recall bias, compared to data from e.g. questionnaires [41]. We did not use the SMS-tracking system to inquire about PA during academic classes at control schools since this could have caused measurement reactivity. Instead, a short questionnaire was distributed to teachers both at intervention and control schools asking to what extent they would perceive themselves as having initiated PA in academic classes during the intervention period.

A custom made mathematics test was chosen, because prior to the study it was found that the paper-based standardized test result [28] did not show normal distribution since a high number of children were polarized and ended in the very low or the very high end of the scale. However, results from the test chosen had a high correlation with the standardized test (correlation coefficient of 0.87 (p < 0.001)).

Also worth mentioning is the geographical diverse distribution of schools in the study. The 14 schools were distributed in all five main regions of Denmark and contained both rural and urban school districts, and small and large schools. As the randomization was stratified only by level of the students obtained final examination grades in the school, most large schools ended up as control schools, which unfortunately resulted in an uneven number of students in the intervention and the control group.

Most of the recruited schools were participating in a primary-school project about PA and health [25], which can be considered both a strength and a weakness of the study. Positively, the engagement of schools could be considerable, because the involvement in the other project shows that both managers and employees had an interest in working with PA as an intervention element. Furthermore, as the other project was aimed at primary school, none of the students in 6^th^ and 7^th^ grade or their primary teachers have been involved in any PA intervention prior to the start of the present study. On the other hand, this selection of schools already motivated for PA can introduce a possible limited potential for change, as also the control schools have the awareness of possible benefits of PA during the school day. In that regard it is possible that initiatives not assessed by the research group can have confounded the results. Other inherent limitations of the present study include the relatively short term intervention; however, training studies have found effects of PA on executive function in shorter term interventions e.g. [23, 38].

We only included one measure of academic performance (mathematics). Conclusions on overall academic achievement are therefore not possible and potential benefits of the intervention in other subjects cannot be assessed.

This manuscript provides a description of the study design, data collection procedures and methods of a cluster-randomized controlled study on children in 6^th^ and 7^th^ grades, implementing a multicomponent PA intervention. This study can add significantly to the scientific knowledge concerning school-based PA interventions including possible effects on cognitive function and academic skills.

## Abbreviations

PA: Physical activity
SMS: Short message service
msec: Milliseconds
RT: Reaction time
SES: Socio economic status
SD: Standard deviation.
